# Clinical impact of rebiopsy among patients with epidermal growth factor receptor‐mutant lung adenocarcinoma in a real‐world clinical setting

**DOI:** 10.1111/1759-7714.13857

**Published:** 2021-02-02

**Authors:** Yunha Nam, Ho Cheol Kim, Young‐Chul Kim, Seung Hun Jang, Kye Young Lee, Shin Yup Lee, Sang Hoon Lee, Sung Yong Lee, Seong Hoon Yoon, Jeong‐Seon Ryu, Tae Won Jang, Yoon Soo Chang, Seung Joon Kim, Chan Kwon Park, Jeong Eun Lee, Chi Young Jung, Chang‐Min Choi

**Affiliations:** ^1^ Department of Pulmonary and Critical Care Medicine, Asan Medical Center University of Ulsan College of Medicine Seoul South Korea; ^2^ Department of Internal Medicine Chonnam National University Medical School, and Chonnam National University Hwasun Hospital Hwasun South Korea; ^3^ Department of Pulmonary Allergy and Critical Care Medicine, Hallym University Sacred Heart Hospital Anyang South Korea; ^4^ Department of Pulmonary Medicine Konkuk University School of Medicine Seoul South Korea; ^5^ Department of Internal Medicine Kyungpook National University, School of Medicine Daegu South Korea; ^6^ Division of Pulmonology, Department of Internal Medicine Severance Hospital, Yonsei University College of Medicine Seoul South Korea; ^7^ Division of Pulmonary, Allergy, and Critical Care Medicine, Department of Internal Medicine Korea University Guro Hospital, Korea University College of Medicine Seoul South Korea; ^8^ Department of Pulmonology and Allergy Pusan National University Yangsan Hospital Yangsan South Korea; ^9^ Department of Internal Medicine Inha University Hospital Incheon South Korea; ^10^ Department of Internal Medicine Kosin University Medical College Pusan South Korea; ^11^ Department of Internal Medicine Yonsei University College of Medicine Seoul South Korea; ^12^ Division of Pulmonology, Department of Internal Medicine The Cancer Research Institute, College of Medicine, The Catholic University of Korea Seoul South Korea; ^13^ Division of Pulmonary and Critical Care Medicine, Department of Internal Medicine Yeouido St. Mary's Hospital, College of Medicine, The Catholic University of Korea Seoul South Korea; ^14^ Division of Pulmonology, Department of Internal Medicine College of Medicine, Chungnam National University Daejeon South Korea; ^15^ Division of Pulmonary and Critical Care Medicine, Department of Internal Medicine Daegu Catholic University Medical Center Daegu South Korea; ^16^ Department of Oncology Asan Medical Center, University of Ulsan College of Medicine Seoul South Korea

**Keywords:** lung cancer, EGFR‐TKI, acquired resistance, T790M, rebiopsy

## Abstract

**Background:**

In this study, we investigated the risk factors of acquired T790M mutation among patients with lung adenocarcinoma with epidermal growth factor receptor (EGFR) tyrosine mutation who were treated with EGFR‐tyrosine kinase inhibitors (TKIs). The aim was to identify the clinical impact of rebiopsy.

**Methods:**

This multicenter, retrospective cohort study was conducted in South Korea from January 2007 to June 2017. Patients with adenocarcinoma with *EGFR* mutation who underwent rebiopsy and were treated with EGFR‐TKIs were included.

**Results:**

Of a total of 352 patients, T790M mutation was identified in 156 (41.9%) at the time of rebiopsy. The median duration from initial biopsy to rebiopsy was 17 months. Univariate logistic regression analysis revealed associations of exon 19 deletion (odds ratio [OR], 1.643; *p* = 0.026), absence of L858R (OR, 0.627; *p* = 0.042), and previous EGFR‐TKI treatment duration (OR, 1.039; *p* < 0.001) with T790M mutation. Previous EGFR‐TKI treatment duration (OR, 3.580; *p* < 0.001) was independently associated with T790M mutation. A multivariate Cox proportional hazard model revealed that brain metastasis at initial diagnosis (hazard ratio, 1.390; *p* = 0.050) tended to be associated with T790M mutation. Among the patients with T790M mutation at rebiopsy, the osimertinib user group (*n* = 90) had a better one‐year survival (68.7 vs. 58.3%, *p* = 0.048) than the osimertinib nonuser group (*n* = 66).

**Conclusions:**

Rebiopsy might affect the clinical course of patients with *EGFR*‐mutant adenocarcinoma who receive EGFR‐TKIs.

## Graphical abstract

In this multicenter retrospective study, whereas exon 19 deletion, the absence of L858R, previous EGFR‐TKI treatment duration and brain metastasis at initial diagnosis were associated with acquired T790M mutation, a previous EGFR‐TKI treatment duration was independently associated with acquired T790M mutation. However, the acquired T790M mutation group had improved survival compared to the T790M mutation‐negative group. In particular, the osimertinib user group had a better one‐year survival than the nonuser group among patients with acquired T790M mutation.

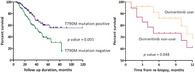

## INTRODUCTION

Lung cancer is still the leading cause of cancer‐related death worldwide.^1^ According to the histological classification, non‐small cell lung cancer (NSCLC) accounts for over 80% of lung cancer cases,^2^ and adenocarcinoma is the most common subtype worldwide.^3^ Although immunotherapy, such as immune checkpoint inhibitors (ICIs), has recently been highlighted,^4^ epidermal growth factor receptor (EGFR) tyrosine kinase inhibitors (TKIs) are recommended as a first‐line therapy for patients with advanced adenocarcinoma with *EGFR* mutation.^5^, ^6^

Unfortunately, acquired resistance occurs in the majority of patients treated with EGFR‐TKIs, with various resistance mechanisms.^7^, ^8^, ^9^ A point mutation in exon 20 (T790M mutation) accounts for over half of the acquired resistance to EGFR‐TKIs,^10^, ^11^ which has led to the development of osimertinib, a third‐generation EGFR‐TKI.^12^ Thus, it is important to identify T790M mutations in patients who are suspected to have acquired resistance to EGFR‐TKIs in a clinical setting. There is growing evidence regarding repeat biopsy.^13^, ^14^, ^15^ However, in many previous studies, the number of patients was relatively small, or the main focus was on feasibility or diagnostic accuracy. In addition, there is a lack of data on the prevalence and clinical characteristics of acquired T790M mutation in a real‐world setting.

Here, we investigated the risk factors of acquired T790M mutation among patients with lung adenocarcinoma with *EGFR* mutation who were treated with EGFR‐TKIs. The intent was to identify the clinical impact of rebiopsy in a real‐world clinical setting.

## METHODS

### Population cohort

**FIGURE 1:**
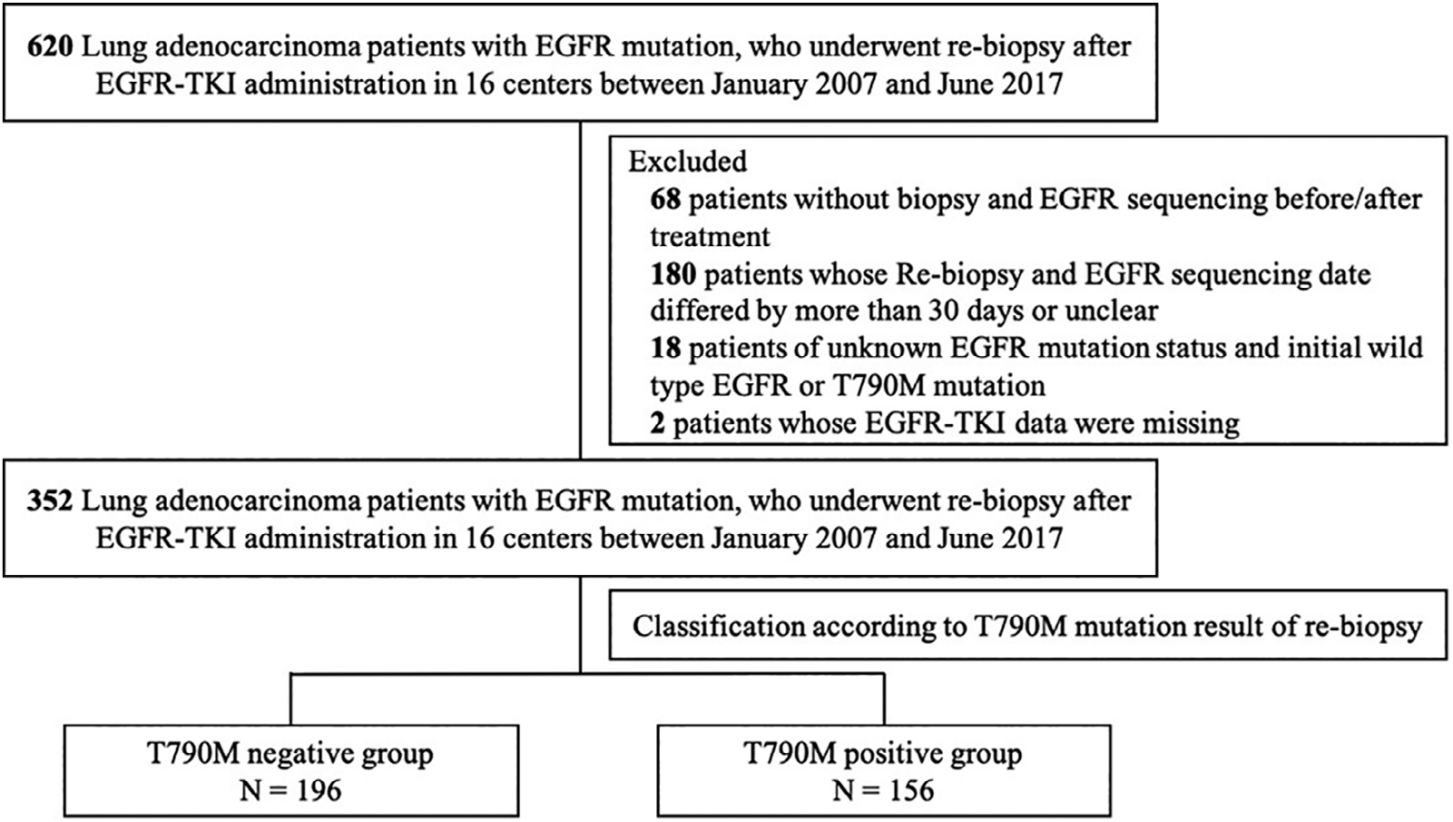
Flow chart of this study

This multicenter, retrospective cohort study involved “the lung cancer patients who were *EGFR* mutation positive in Korea” cohort. Data were gathered from The Korean Academy of Tuberculosis and Respiratory Disease. From January 2007 to June 2017, adenocarcinoma patients with *EGFR* mutation who underwent rebiopsy during the follow‐up period and were treated with EGFR‐TKIs were included in this study. Among the eligible patients, those who did not undergo an *EGFR* mutation test, had an interval of more than one month between biopsy and *EGFR* detection, or whose information about the *EGFR* mutation test was unknown, were excluded. Patients who initially had T790M mutation were also excluded (Figure 1).

A standardized protocol was used to collect data regarding age, gender, smoking history, clinical stage at the time of initial diagnosis, brain metastasis, repeat biopsy performed during the follow‐up period, and treatment information regarding conventional chemotherapy and EGFR‐TKIs. The repeat biopsy information included the date and site of biopsy, histological diagnosis, timing correlation between biopsy and EGFR‐TKI treatment, *EGFR* mutation status, and its detection method. The previous TKI treatment duration was set as the sum from the start date to the end date of each TKI administration. The study protocol was approved by the Institutional Review Board of Asan Medical Center (approval number: 2018‐0092). Requirements for written informed consent were waived due to the retrospective nature of the present study and the use of anonymized patient data.

### Evaluation of *EGFR* mutations

The peptide nucleic acid (PNA)‐mediated PCR clamping method is the gold standard for the detection of *EGFR* mutations. The method uses formalin‐fixed paraffin‐embedded tumor tissue obtained via tissue biopsy. Previously, direct sequencing (DS) methods were commonly used to compare amplified sequences with GenBank EGFR through PCR. Recently, Mutyper methods have been used, in which circulating‐free tumor DNA (ctDNA) is detected in plasma after collecting blood into an EDTA anticoagulation bottle. As this cohort was recruited from multiple institutions, various *EGFR* mutation detection techniques were used. They included direct sequencing and the aforementioned PNA clamping method and Mutyper as mentioned above. We used the PNAClamp EGFR, PANAqPCR, and PANAMutyper *EGFR* mutation detection kits (all from Panagene) to detect the various *EGFR* mutations.

### Statistical analysis

All values are expressed as median and interquartile range (IQR) for continuous variables or percentages for categorical variables. Student's *t*‐test or the Mann‐Whitney U test was used to examine continuous data. A chi‐square test or Fisher's exact test was used to examine the categorical data. Logistic regression and Cox regression analysis were performed to identify the factors affecting T790M mutation. Furthermore, variables with *p* < 0.2 in the univariate analysis were entered into the multivariate models. Survival analysis was performed using Kaplan‐Meier analysis and log‐rank test. IBM SPSS statistics v.20 (IBM, Armonk) was used for analyses. A statistically significant difference was defined as a *p*‐value < 0.05.

## RESULTS

### Patient characteristics

A total of 352 patients with adenocarcinoma and *EGFR* mutations were included in this study. The median follow‐up period was 22 months (interquartile range [IQR]: 15–36 months). The baseline characteristics of patients are summarized in Table 1. The median age was 58 years (IQR: 52–68 years); 34.9% patients were male and 29.0% were ever‐smokers. Most patients (90.9%) initially had stage IV adenocarcinoma and the lungs were the most common initial biopsy site (67.5%). Regarding *EGFR* mutation, exon 19 deletion was the most common (60.8%), followed by L858R (34.7%), L861Q (5.4%), and G719X (2.3%). A total of 80.6% of patients were treated with an EGFR‐TKI. They initially included gefitinib, erlotinib, or afatinib. Approximately 13.4% received cytotoxic chemotherapy.

**TABLE 1:** Baseline and clinical characteristics of the study patients

Characteristics	Total
Number (n)	352
Median age, years	58 [52–68]
Male	130 (34.9)
Ever‐smoker	108 (29.0)
Initial clinical stage	
I	1 (0.3)
II	2 (0.5)
III	6 (1.6)
IV	338 (90.9)
Initial biopsy site	
Lungs	251 (67.5)
Lymph nodes	69 (18.5)
Pleural effusion	13 (3.5)
Others^a^	19 (5.1)
Initial *EGFR* mutation	
Exon 19 del	214 (60.8)
L858R	122 (34.7)
L861Q	19 (5.4)
G719X	8 (2.3)
Others^b^	11 (3.1)
Initial chemotherapy regimen	
Cytotoxic chemotherapy	50 (13.4)
EGFR‐TKI	300 (80.6)
Gefitinib	200 (53.8)
Erlotinib	42 (11.3)
Afatinib	57 (15.3)

Note: Data are presented as mean ± standard deviation, median [interquartile range], or number (%), unless otherwise indicated.

Abbreviations: EGFR, epidermal growth factor receptor; TKI, tyrosine kinase inhibitor.

^a^ Others: Initial biopsy site was the brain in eight cases, the bronchus in two cases, pleura in two cases, liver in two cases, bone in two cases, the pericardial fluid in one case, pericardium in one case, and adrenal gland in one case.

^b^ Others: Four cases were E746_A750del, one case each presented p.D770_n771insG, I706T, exon 20 insertion, E746_T751del, E709G, codon 770, and 745aa.

### Acquired T790M mutation

All eligible patients underwent repeat biopsy during the follow‐up period. The median duration from initial biopsy to rebiopsy was 17 months (IQR 10–29 months). T790M mutation was identified in 156 (41.9%) patients at the time of rebiopsy. The comparison of characteristics according to the T790M mutation status at the time of rebiopsy is shown in Table 2. There were no significant differences in demographics, brain metastasis, rebiopsy site, and initial TKI treatment between the two groups. However, patients with T790M mutation had a longer duration between initial biopsy to rebiopsy (median, 19 vs. 16 months, *p* = 0.008) compared to the patients without T790M mutation. According to the initial EGFR profile, exon 19 deletion was more frequent in patients with T790M mutation (67.3 vs. 55.6%, *p* = 0.028), whereas L858R was less frequent in the T790M mutation group (28.8 vs. 39.3%, *p* = 0.043).

**TABLE 2:** Baseline and clinical characteristics of patients according to T790M mutation at the time of rebiopsy

Characteristics	Total	T790M positive	T790M negative	*p*‐value
Patient numbers	352	156 (41.9)	196 (52.7)	
Median age, years	58 [52–68]	58 [50–68]	59 [52–69]	0.318
Male	130 (36.9)	59 (37.8)	71 (36.2)	0.758
Ever‐smoker	108 (30.7)	49 (31.4)	59 (30.1)	0.384
Duration from initial biopsy to rebiopsy [median]	17 [10–29]	19 [13–30]	16 [9–28]	0.008
Brain metastasis	126 (35.8)	57 (36.5)	69 (35.2)	0.952
Rebiopsy site				0.369
Lungs	178 (50.6)	75 (48.1)	103 (52.6)	
Lymph nodes	75 (21.3)	32 (20.5)	43 (21.9)	
BAL	5 (1.4)	5 (3.2)	0 (0)	
Pleural fluid	26 (7.4)	12 (7.7)	14 (7.1)	
Others^a^	68 (19.3)	32 (20.5)	36 (18.4)	
Initial EGFR profile				
Exon 19 del	214 (60.8)	105 (67.3)	109 (55.6)	0.028
L858R	122 (34.7)	45 (28.8)	77 (39.3)	0.043
L861Q	19 (5.4)	7 (4.5)	12 (6.1)	0.637
G719X	8 (2.3)	2 (1.3)	6 (3.1)	0.309
Others^b^	11 (3.1)	5 (3.2)	6 (3.1)	>0.999
Initial TKI regimen				0.460
First‐generation	292 (83.0)	131 (37.2)	161 (45.7)	
Second‐generation	60 (17.0)	25 (7.1)	35 (9.9)	

Note: Data are presented as mean ± standard deviation, median [interquartile range], or number (%) unless otherwise indicated.

Abbreviations: BAL, bronchoalveolar lavage; EGFR, epidermal growth factor receptor; TKI, tyrosine kinase inhibitor.

^a^ Others:20 cases at liver, nine cases at pleura, 10 cases at bone, five cases each for ascitic fluid and cerebrospinal fluid, three cases at adrenal gland, two cases each at bronchus, brain, chest wall, pericardium, and pericardial fluid, one case each at breast, blood, kidney, large intestine, nasal cavity, and skin

^b^ Others: Four cases were E746_A750del, and one case each presented p.D770_n771insG, I706T, exon 20 insertion, E746_T751del, E709G, codon 770, and 745aa.

Treatment information according to T790M mutation status at the time of rebiopsy is summarized in Table S1. Although there were no significant differences in number, type, and the best response to EGFR‐TKIs between the two groups, patients with T790M mutation had a longer EGFR‐TKI treatment duration (median 15 vs. 11 months, *p* < 0.001) compared to patients without T790M mutation. Among the 156 patients with T790M mutation, 66 were treated with the third‐generation TKI, osimertinib. In 90 patients who were not treated with osimertinib after repeat biopsy, 29 (32.2%) received conservative management, 24 (26.7%) participated in clinical trials, 18 (20%) received cytotoxic chemotherapy, and 19 (21.1%) received different types of EGFR‐TKI.

### Risk factors of acquired T790M mutation

To identify the risk factors of acquired T790M mutation, we performed logistic regression analysis (Table 3) and Cox regression analysis (Table 4). Univariate logistic analysis showed that the presence of exon 19 deletion (odds ratio [OR], 1.643; 95% CI: 1.061–2.545; *p* = 0.026) and the absence of L858R (OR, 0.627; 95% CI, 0.400–0.982; *p* = 0.042) were associated with T790M mutation. The previous EGFR‐TKI treatment duration (OR, 1.039; 95% CI: 1.019–1.058; *p* < 0.001) was also associated with T790M mutation. Multivariate logistic analysis showed that previous EGFR‐TKI treatment duration (OR, 3.580; 95% CI: 2.257–5.678; *p* < 0.001) was the only factor independently associated with T790M mutation. In addition, the presence of exon 19 deletion displayed a trend toward association with T790M mutation (OR, 1.509; 95% CI: 0.955–2.386; *p* = 0.078).

**TABLE 3:** Risk factors for T790M mutation in patients with *EGFR* mutation assessed using logistic regression analysis

Parameter	Hazard ratio	95% CI	*p*‐value
Univariate analysis			
Age	0.989	0.970–1.008	0.257
Male	0.934	0.604–1.443	0.758
Ever‐smoker	0.241	0.027–2.131	0.201
Brain metastasis	1.039	0.801–1.347	0.772
Same biopsy site as initial biopsy	1.069	0.702–1.628	0.757
Initial EGFR profile			
Exon 19 del	1.643	1.061–2.545	0.026
L858R	0.627	0.400–0.982	0.042
L861Q	0.720	0.277–1.875	0.502
G719X	0.411	0.082–2.066	0.281
Other	1.049	0.314–3.502	0.939
Best response to TKI treatment (disease progression)	0.272	0.076–0.971	0.045
Previous EGFR‐TKI days	1.039	1.019–1.058	< 0.001
Multivariate analysis			
Initial exon 19 del	1.509	0.955–2.386	0.078
Best response to TKI treatment (disease progression)	3.089	0.817–11.688	0.097
Previous EGFR‐TKI days	3.580	2.257–5.678	<0.001

Abbreviations: EGFR, epidermal growth factor receptor; TKI, tyrosine kinase inhibitor.

**TABLE 4:** Risk factors for T790M mutation in patients with *EGFR* mutation assessed using Cox regression analysis

Parameter	Hazard ratio	95% CI	*p*‐value
Univariate analysis			
Age	1.006	0.991–1.021	0.425
Male	0.933	0.793–1.098	0.401
Ever‐smoker	0.550	0.148–2.042	0.372
Brain metastasis	1.439	1.035–2.001	0.030
Same biopsy site as initial biopsy	1.108	0.808–1.519	0.524
Initial EGFR profile			
Exon 19 del	1.081	0.772–1.515	0.649
L858R	1.040	0.734–1.474	0.823
L861Q	1.043	0.488–2.230	0.914
G719X	0.548	0.135–2.214	0.398
Other	0.616	0.226–1.680	0.344
Best response to TKI treatment (disease progression)	0.444	0.141–1.394	0.164
Previous EGFR‐TKI days	0.836	0.578–1.210	0.343
Multivariable analysis			
Brain metastasis	1.390	0.999–1.933	0.050

Abbreviations: EGFR, epidermal growth factor receptor; TKI, tyrosine kinase inhibitor.

The univariate Cox proportional hazard model showed that only brain metastasis at the initial biopsy was associated with an acquired T790M mutation (hazard ratio [HR], 1.439; 95% CI: 1.035–2.001; *p* = 0.030). In the multivariate Cox proportional hazard model, brain metastasis tended to be associated with T790M mutation. Patients with brain metastasis had a shorter duration between initial biopsy and rebiopsy (median, 15 vs. 19 months; *p* = 0.003) compared to patients without brain metastasis (Table S2). Patients with brain metastasis also had a shorter EGFR‐TKI treatment duration (median, 12 vs. 14 months; *p* = 0.009) than patients without brain metastasis.

### Survival analysis

**FIGURE 2:**
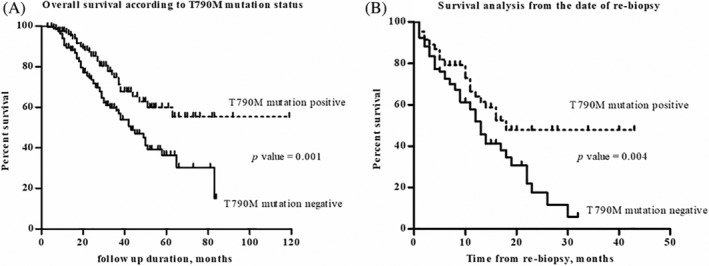
(a) Overall survival according to T790M mutation status. (b) Survival analysis from the date of rebiopsy

The T790M mutation positive group (*n* = 156) had an improved survival (median survival period: 43 months vs. not reached, *p* = 0.001) than the T790M mutation negative group (*n* = 196, Figure 2(a)). Even if the start date was the date of rebiopsy for the survival analysis, the T790M mutation positive group still had an improved survival (median survival period: 12 vs. 18 months, *p* = 0.004) than the T790M mutation negative group (Figure 2(b)).

**FIGURE 3:**
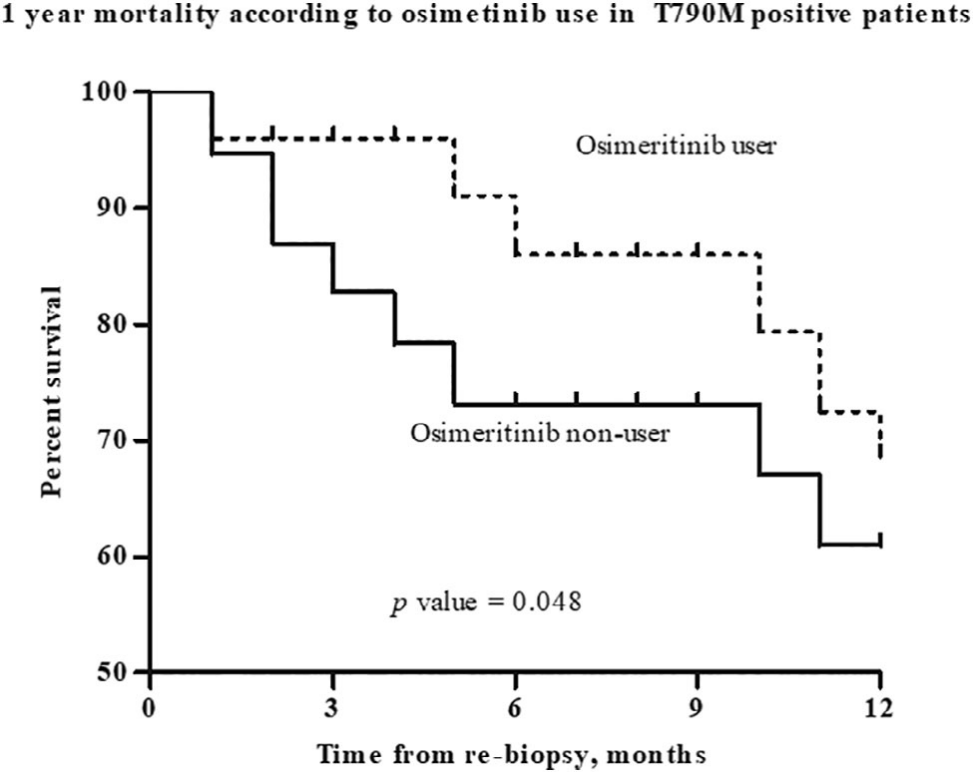
One‐year mortality according to osimertinib use in T790M positive patients

Among the patients with T790M mutation at the time of rebiopsy, the osimertinib user group (*n* = 90) had a better one‐year survival (68.7 vs. 58.3%, *p* = 0.048) than the osimertinib nonuser group (*n* = 66, Figure 3). The baseline and clinical characteristics in patients with T790M mutation treated with osimertinib are shown in Table S3. There were no significant differences in the clinical characteristics between the two groups.

## DISCUSSION

In this study, acquired T790M mutation was identified at the time of rebiopsy in approximately 40% of patients with *EGFR*‐mutant adenocarcinoma who were treated with EGFR‐TKI. Exon 19 deletion, absence of L858R, previous EGFR‐TKI treatment duration, and brain metastasis at initial diagnosis were associated with acquired T790M mutation. Previous EGFR‐TKI treatment duration was also independently associated with acquired T790M mutation. However, the acquired T790M mutation group had improved survival compared to the T790M mutation negative group. In particular, the osimertinib user group had a better one‐year survival than the osimertinib nonuser group among patients with acquired T790M mutation. These findings suggest that rebiopsy might affect the clinical course of patients with *EGFR*‐mutant adenocarcinoma.

Acquired T790M mutation is regarded as one of the most important resistance mechanisms and clinical responses during EGFR‐TKI treatment.^16^, ^17^ Among our cohort, acquired T790M mutation was identified in 41.9% of patients, which is comparable to previous studies.^7^, ^18^, ^19^ In a study of 37 patients with EGFR‐TKI‐resistant NSCLC, 18 patients (49%) showed acquired T790M mutation.^7^ In another study involving 57 *EGFR* mutation‐positive NSCLC patients who had undergone repeat biopsy after failure of treatment with a first‐ or second‐generation EGFR‐TKI, acquired T790M mutation was identified in 27 patients (47%).^18^ A review identified T790M mutation in 51.9% of study patients suspected of having TKI resistance.^19^ However, in most previous studies, including this study, the frequency of acquired T790M mutation was investigated only in patients that received rebiopsy, which might induce selection bias. Further studies are needed to ascertain the exact frequency of T790M mutation among patients treated with EGFR‐TKI.

Several factors are presently associated with the development of acquired T790M mutation. The presence of exon 19 deletion, absence of L858R, and previous EGFR‐TKI treatment duration were associated with T790M mutation in logistic regression analysis. Brain metastasis at initial biopsy was associated with acquired T790M mutation in the Cox regression analysis. Previous studies support our results.^20^, ^21^, ^22^, ^23^ An analysis of 25 studies involving 1,770 patients found that acquired T790M mutation (after treatment with EGFR‐TKIs) was more common in patients with exon 19 deletion (53% vs. 36%, *p* < 0.001) than in patients with L858R mutation.^20^ Likewise, a study involving 111 patients with adenocarcinoma with *EGFR* mutation found that exon 19 deletion was independently associated with acquired T790M mutation (*p* = 0.003) in a multivariable analysis.^21^ In the present study, initial EGFR‐TKI treatment (first‐ or second‐generation TKIs) was not associated with acquired T790M mutation which is comparable with the results of a previous study.^24^ A study involving 233 NSCLC patients with *EGFR* mutation reported that the acquired resistance rate of T790M mutation was not different between EGFR‐TKI types (42.9% of gefitinib users, 45.7% of erlotinib users, and 45.3% of afatinib users).^24^ However, other authors have previously described a higher incidence of T790M mutation in patients who received gefitinib or erlotinib (OR 7.1, *p* < 0.001) compared with patients who received afatinib.^25^ More large‐scale studies are needed to reveal if there is an association between acquired T790M mutation and EGFR‐TKI type. Using a logistic regression analysis, we found that previous EGFR‐TKI treatment duration was independently associated with T790M mutation. Similarly, in a study involving 73 NSCLC patients treated with EGFR‐TKIs, Matsuo et al.^26^ reported that a longer duration of treatment was independently associated with T790M mutation in a multivariate logistic analysis (≤10 vs. >10 months, OR 0.09, *p* < 0.001). These findings suggest that close monitoring of T790M mutation is needed, especially in long‐term users of EGFR‐TKIs. Brain metastasis is related to *EGFR* mutations in patients with lung adenocarcinoma and T790M mutation.^22^, ^23^ In a previous meta‐analysis of 22 studies that included NSCLC patients with or without *EGFR* mutations, the presence of an *EGFR* mutation was associated with a markedly higher incidence of subsequent brain metastasis and an increasing trend in the incidence of initial brain metastasis.^22^ Another study with 212 NSCLC patients reported that T790M mutation was more common in patients with brain metastasis (*n* = 12/40, 30%, *p* < 0.01).^23^ Presently, the duration from initial biopsy to rebiopsy was significantly longer in patients without brain metastasis than in patients with brain metastasis (median 15 vs. 19 months, *p* = 0.003). Brain metastasis at initial diagnosis was associated with acquired T790M mutation. These findings reveal the importance of monitoring for acquired T790M mutation, especially in patients with brain metastasis.

Several previous studies have suggested that patients with acquired T790M mutation would have a favorable clinical course compared to patients with acquired resistance lacking the T790M mutation.^16^, ^21^, ^27^ In one of these studies, which involved 93 patients with *EGFR*‐mutant lung adenocarcinoma, patients with T790M (*n* = 58, 62%) had better post‐progression survival (median survival, 19 vs. 12 months; *p* = 0.036) than T790M‐negative patients.^16^ In another study of 111 patients with *EGFR*‐mutant lung adenocarcinoma, the acquired T790M‐mutant group (*n* = 58, 52.3%) had a significantly longer overall survival (*p* = 0.010) compared to patients without T790M mutation.^21^ Although the exact mechanisms underlying these results are unclear, preclinical data has led to the conclusion that *EGFR*‐mutant cell lines with acquired T790M mutation feature a more indolent growth than the parental cell lines.^27^ Osimertinib is an irreversible EGFR‐TKI that selectively inhibits both EGFR‐TKI sensitizing mutations and T790M‐resistance mutations. The efficacy of osimertinib has been previously described.^12^, ^28^, ^29^ As a representative example, in the AURA study involving 419 patients with T790M‐positive advanced NSCLC, the osimertinib group showed longer progression‐free survival (10.1 vs. 4.4 months, *p* < 0.001) compared to patients treated with platinum therapy plus pemetrexed.^29^ Osimertinib treatment also showed clinical benefits in T790M‐negative patients in a study involving 199 lung adenocarcinoma patients diagnosed with *EGFR* mutations.^28^ In the present study, while 42.3% of the T790M‐positive group received osimertinib, it was only administered to 7.7% of the T790M negative group. This may have reflected insurance coverage in South Korea. The difference in osimertinib usage between the T790M positive and negative groups indicate that treatment might beneficially affect survival.

There were several limitations in this study. First, it was retrospective and only patients who underwent rebiopsy were included, and this may have resulted in selection bias. Second, the study included only Korean patients which might limit the generalizability of the results. However, the study was performed at several centers, and the baseline and clinical characteristics of the study patients were comparable with those in previous studies.^15^, ^18^, ^19^ Third, the initial biopsy and rebiopsy sites differed in 175/352 (49.7%) patients, which might influence the T790M mutation status. Fourth, data concerning other important resistance mechanisms to TKIs, such as human EGFR 2 amplification, mesenchymal‐epithelial transition factor amplification, and epithelial‐mesenchymal transition, were not collected during the study period.^30^ Finally, although we included a relatively large number of patients, the numbers were not adequate to ascertain meaningful comparisons within subgroups.

In conclusion, rebiopsy might influence the clinical course in adenocarcinoma patients with *EGFR* mutation who received EGFR‐TKI. Further prospective studies involving larger populations with different ethnic populations are warranted.

## CONFLICT OF INTEREST

The authors have no conflict of interest to disclose.

## Supporting information


**Table S1** Treatment information of the patients according to T790M mutation at the time of re‐biopsy.
**Table S2** Baseline and clinical characteristics of patients according to initial brain metastasis.
**Table S3** Comparison of baseline characteristics of osimertinib users among T790M positive patients.
