# Supplementary material for: Clinical impact of rebiopsy among patients with epidermal growth factor receptor‐mutant lung adenocarcinoma in a real‐world clinical setting
Source: Thorac Cancer. 2021 Feb 2;12(6):890–8. doi: 10.1111/1759-7714.13857 (PMC7952806; doi:10.1111/1759-7714.13857)
Supplement: Supplementary file 1 — Table S1 Treatment information of the patients according to T790M mutation at the time of re‐biopsy. Table S2 Baseline and clinical characteristics of patients according to initial brain metastasis. Table S3 Comparison of baseline characteristics of osimertinib users among T790M positive patients. [file TCA-12-890-s001.docx]

**Table S1** Treatment information of the patients according to T790M mutation at the time of re-biopsy.

| Characteristics | Total | T790M positive | T790M negative | P-value |
| --- | --- | --- | --- | --- |
| Previous EGFR-TKI number |  |  |  | 0.377 |
| 1 | 317 (90.1) | 138 (88.5) | 179 (91.3) |  |
| ≥ 2 | 35 (9.9) | 18 (11.5) | 17 (8.7) |  |
| Best response of TKI treatment | |  |  | 0.078 |
| CR | 1 (0.3) | 1 (0.6) | 0 (0) |  |
| PR | 238 (67.6) | 111 (71.2) | 127 (64.8) |  |
| SD | 92 (26.1) | 40 (25.6) | 52 (26.5) |  |
| PD | 16 (4.5) | 3 (1.9) | 13 (6.6) |  |
| EGFR-TKI treatment duration (months) | 12 [7–16] | 15 [11–24] | 11 [6–18] | < 0.001 |

Data are presented as mean ± standard deviation, median [interquartile range], or number (%) unless otherwise indicated. EGFR: Epidermal growth factor receptor; TKI: Tyrosine kinase inhibitor; CR: complete response; PR: partial response; SD: stable disease; PD: progressive disease

**Table S2** Baseline and clinical characteristics of patients according to initial brain metastasis.

| Characteristics | Total | Brain metastasis | No metastasis | P-value |
| --- | --- | --- | --- | --- |
| Patients number (n) | 350 | 126 (36.0) | 224 (64.0) |  |
| Age [median] | 58 [52–68] | 57 [51–68] | 58 [52–68] | 0.325 |
| Male | 130 (37.1) | 48 (38.1) | 82 (36.6) | 0.818 |
| Ever-smoker | 108 (31.4) | 28 (31.1) | 70 (31.5) | > 0.999 |
| Duration from initial biopsy to re-biopsy (median) | 17 [10–29] | 15 [9–24.25] | 19 [11–31] | 0.003 |
| Initial EGFR profile |  |  |  |  |
| Exon 19 del | 213 (60.9) | 82 (65.1) | 131 (58.5) | 0.254 |
| L858R | 121 (34.6) | 41 (32.5) | 80 (35.7) | 0.561 |
| G719X | 8 (2.3) | 3 (2.4) | 5 (2.2) | 1.000 |
| L681Q | 19 (5.4) | 5 (4.0) | 14 (6.3) | 0.626 |
| Other | 11 (3.1) | 3 (2.4) | 8 (3.6) | 0.802 |
| EGFR-TKI treatment duration, (months) | 12 [7–16] | 12 [7–16] | 14 [9–23] | 0.009 |

Data are presented as mean ± standard deviation, median [interquartile range], or number (%) unless otherwise indicated. EGFR: Epidermal growth factor receptor; TKI: Tyrosine kinase inhibitor

**Table S3** Comparison of baseline characteristics of osimertinib users among T790M positive patients.

| Characteristics | Total | Osimertinib user | Osimertinib non-user | P-value |
| --- | --- | --- | --- | --- |
| Patients number (n) | 156 | 66 (42.3) | 90 (57.7) |  |
| Age [median] | 58 [50–68] | 56.5 [50.7–67.2] | 59 [50.0–68.0] | 0.689 |
| Male | 59 (37.8) | 25 (37.9) | 34 (37.8) | > 0.999 |
| Ever-smoker | 49 (31.4) | 20 (30.3) | 29 (32.2) | 0.661 |
| Brain metastasis | 57 (36.5) | 22 (33.3) | 35 (38.9) | 0.516 |
| Clinical stage at diagnosis | |  |  | 0.528 |
| II | 1 (0.6) | 0 (0) | 1 (1.1) |  |
| III | 3 (1.9) | 2 (3.0) | 1 (1.1) |  |
| IV | 149 (95.5) | 62 (93.9) | 87 (96.7) |  |
| Previous EGFR-TKI number | |  |  | 0.436 |
| 1 | 138 (89.0) | 56 (89.2) | 82 (91.1) |  |
| 2 | 17 (11.0) | 9 (13.8) | 8 (8.9) |  |
| Previous EGFR-TKI |  |  |  |  |
| Gefitinib | 107 (68.6) | 39 (59.1) | 68 (75.6) | 0.036 |
| Erlotinib | 35 (22.4) | 16 (24.2) | 19 (21.1) | 0.700 |
| Afatinib | 29 (18.6) | 20 (30.3) | 9 (10.0) | 0.002 |

Data are presented as mean ± standard deviation, median [interquartile range], or number (%) unless otherwise indicated
